# Social and Economic Factors Associated With Subthreshold and Major Depressive Episode in University Students During the COVID-19 Pandemic

**DOI:** 10.3389/fpubh.2022.893483

**Published:** 2022-05-18

**Authors:** Álvaro I. Langer, Marcelo A. Crockett, Mariori Bravo-Contreras, Carolina Carrillo-Naipayan, Matías Chaura-Marió, Bárbara Gómez-Curumilla, Claudia Henríquez-Pacheco, Rodrigo C. Vergara, Jorge Santander, Zayra Antúnez, Tomas Baader

**Affiliations:** ^1^Mind-Body Lab, Institute of Psychological Studies, Austral University, Valdivia, Chile; ^2^Millennium Nucleus to Improve the Mental Health of Adolescents and Youths, Imhay, Santiago, Chile; ^3^Millennium Institute for Research on Depression and Personality, MIDAP, Santiago, Chile; ^4^Escuela de Salud Pública, Facultad de Medicina, Universidad de Chile, Santiago, Chile; ^5^Faculty of Medicine, School of Psychology, Austral University, Valdivia, Chile; ^6^Departamento de Kinesiología, Facultad de Artes y Educación Física, Universidad Metropolitana de Ciencias de la Educación, Santiago, Chile; ^7^Centro Nacional de Inteligencia Artificial CENIA, Santiago, Chile; ^8^Faculty of Medicine, Institute of Clinical Neuroscience, Austral University, Valdivia, Chile; ^9^Faculty of Medicine, Institute of Psychological Studies, Austral University, Valdivia, Chile; ^10^Alianza Chilena Contra la Depresión y el Suicidio, Valdivia, Chile

**Keywords:** depression, subthreshold depression, socioeconomic, college students, social support

## Abstract

Depression is one of the most frequent mental health disorders in college students and variations according to social and economic factors have been reported, however, whether social and economic variations also exist in subthreshold depression is still unknown, especially during the COVID-19 pandemic. The aim of this study was to estimate the prevalence of subthreshold depressive episode (SDE) and major depressive episode (MDE) and to examine the association between social and economic factors with SDE and MDE in undergraduate students during the COVID-19 pandemic. The participants were 1,577 college students from a university in the south of Chile (64.6% females, 22 years old on average). The participants took an online survey in November 2020 which collected information about social and economic variables, depressive symptoms, and perceived social support. Bivariate and multinomial logistic regression analysis were used. The results showed a high prevalence of SDE (14.3%) and MDE (32.3%) in the sample. Belonging to a social group and perceiving positive social support were the only variables examined that were associated with SDE. Instead, female sex, poorer quintiles, living with other relatives but not parents, economic difficulties due to the pandemic, being a parent, and perceiving positive social support were associated with MDE. Subthreshold and threshold depressive symptoms are frequent in college students, and associations with social and economic factors differ according to the level of such symptoms. These results should be considered in the development of tailored preventive and early interventions for depression in college students.

## Introduction

Depressive disorders in youth are an important public health concern, ranking fourth among the leading causes of disability-adjusted life-years in the 10–24 age group (1). In college students, depression is one of the most frequent mental health disorders, with a weighted mean prevalence of 30.6%, higher than in the general population (2). Recent data from college students from eight countries showed a lifetime prevalence of major depressive episode (MDE) of 21.2% and a 12-month prevalence of 18.5% (3).

Subthreshold depression is characterized by the presence of clinically significant depressive symptoms, which fall short of the criteria for major depressive disorder (4). There is still no consensus in the literature on the definition of subthreshold depression, for example, regarding the impairment, number, and duration of symptoms (4–6). Various concepts have been used to refer to the same phenomenon (e.g., subsyndromal, subclinical, minor, or mild depression) (5, 6). Despite the differences in definition, it has been observed that people with subthreshold depression possess higher chances of developing major depressive disorder (4, 7) as well as other psychiatric disorders such as anxiety or disruptive disorders (8) and suicidal ideation (9). In the general population, subthreshold depression has been associated with an increased risk of mortality (10) and has considerable economic costs, approaching those of major depression (11). Although subthreshold depression is less severe than major depression, its high prevalence makes its impact on public health an important concern (10, 12).

Regarding the social and economic factors associated with depression in the university population, it has been reported that maintaining positive family relationships, having the possibility of sharing problems with others (13), enjoying strong social support (14), and not having problems with friends (15) are negatively associated with depression. In contrast, gender, economic variables, age, and ethnicity show inconsistent results. Some studies report that women show higher levels of depression (15–18), while other studies have found no gender differences (13, 19–22). Similarly, some studies have found that students from families with a lower income (19, 20), lower socioeconomic status (15), lower parental education (20), housing problems, and larger family size (16) have more depressive symptoms. In contrast, other studies have not found any differences by economic income (13, 15, 22) or parental education (15). Regarding ethnicity, Liu et al. (17) found lower odds of depression in Hispanic, Black, and Asian students, whereas other studies have not found an association between ethnicity and depression (20). Regarding age, studies have reported higher levels of depression in older students (17, 20, 22), while others have observed no differences by age (15, 19, 21). Differences in the measurement of depression could partially explain the above inconsistencies, however, some differences may be related to the level of depressive symptoms, such that some social and economic variables could have a differential impact according to the severity of depression, which may not be captured in previous studies.

In this context, the COVID-19 pandemic has had a significant impact on the mental health of the population, especially adolescents and young people (23). Depression symptoms, anxiety, and sleeping issues have been shown to be the most frequent in university students (24, 25). Specifically for depression, recent meta-analyses indicate a prevalence of 30 to 37% among university students (24, 26–28). Also, differences in prevalence have been found in connection with geographical area, financial situation, and living arrangements (24). Inconsistent results have been found regarding the sex/gender variable (27). In addition, the month of data collection and geographical region were determined to be significant moderators of the prevalence (27). Perceived social support, one of the most frequently studied variables, exhibits an inverse association with depression among students (e.g., (29)). Furthermore, the effects of the pandemic have also been found to increase subthreshold depression in the general population (30) and children (31). To the best of our knowledge, no data are available on subthreshold depression in college students during the pandemic.

In this regard, to understand the impact of COVID-19 on the mental health of university students, authors have suggested exploring its intensity in connection with social and economic factors in various geographical areas (24). Thus, the aim of this study was to estimate the prevalence of subthreshold depressive episode (SDE) and major depressive episode and to examine the association between social and economic factors with SDE and MDE in undergraduate students during the COVID-19 pandemic.

## Methods

### Participants

All undergraduate students of the Universidad Austral de Chile were invited to participate (*N* = 17,720), of whom 1,850 took the online survey (response rate = 10.4%). Students who were not currently taking courses and who did not have complete information on the study variables were excluded from the sample (*n* = 273), reducing the number of participants to 1,577 undergraduate students. The sample consisted of 1,018 females (64.6%) and 559 males (35.4%) with a mean age of 22.0 years (*SD* = 3.1, range = 17–49).

### Measures

Patient Health Questionnaire (PHQ-9) (32). The PHQ-9 is a 9-item self-report instrument that assesses depressive symptoms according to criteria set out in the Diagnostic and Statistical Manual of Mental Disorders-IV. It has a 4-point ordinal response scale ranging from 0 = not at all to 3 = nearly every day. For this study, the Spanish-language version for the Chilean population performed by Baader et al. (33) was used, which has shown adequate levels of reliability and concurrent validity with other measures of depression. In the sample, the PHQ-9 scores had an internal consistency of α = 0.88. The algorithm used in previous studies (34, 35), which resembles the diagnostic criteria for major depressive disorder of the DSM-5 (36), was used to determine the presence of SDE and MDE. Operationally, SDE diagnosis was assigned to students who responded more than half the days (2) and nearly every day (3) in no less than two and no more than four items of the PHQ-9, of which at least one should be item 1 (depressed mood) or 2 (anhedonia), the core symptoms of depression. MDE diagnosis was assigned to students who responded more than half the days (2) and nearly every day (3) in five or more items of the PHQ-9, of which at least one should be item 1 or 2. For both diagnosis criteria, SDE and MDE, item 9 was considered present if the respondent's answer ranged from several days (1) to nearly every day (3), according to the diagnostic algorithm of the instructional manual of the PHQ-9 (37).

The Functional Social Support Questionnaire (Duke-UNC-11) (38). This is an 11-item self-report instrument that assesses perceived social support in two dimensions: confidant support (the possibility to discuss and share important matters with a confidant) and affective support (such as emotional support and caring). It has a 5-point ordinal response scale ranging from 1 = as much as I would like to 5 = much less than I would like. In this study, the total score was used, which is obtained by adding up the scores for each item and which ranges from 11 to 55 points. Lower scores indicate poorer perceived social support. The Spanish-language version adapted for the Chilean population was used (39). The Duke-UNC scores had an internal consistency of α = 0.90 in this sample.

Social and Economic Variables. In addition to the instruments mentioned above, a self-report questionnaire was included to collect information on sociodemographic, social, and economic variables. The variables included were sex (0 = male, 1 = female), age (in years, recoded as 1 = 17–19, 2 = 20–24, and 3 = 25 or more), indigenous ethnicity (0 = no, 1 = yes), lives with (1 = mother, father, or both, 2 = other family members, 3 = other), parenthood (0 = no, 1 = yes), economic quintile (from I to V), economic difficulties due to the COVID-19 pandemic (0 = no, 1 = yes), inadequate study conditions (0 = no, 1 = yes), and social group membership (e.g., volunteer, religious, sports, political or artistic group, among others) (0 = no, 1 = yes). Economic quintile is the distribution of per capita family income of the population divided into five equal parts. Quintile I is composed of the bottom 20% of the population in terms of income, while quintile V is composed of the top 20% (40).

### Procedure

The study was approved by the Scientific Ethical Committee on Human Research of the Faculty of Medicine of the Universidad Austral de Chile. The survey was administered during November 2020, which coincided with the beginning of a quarantine period in the city. Students gave their informed consent to participate in the study. The survey was conducted online through the University's educational platform. After completing the survey, students received information about their results in an automatically generated report, along with recommendations for improving their mental health and information about mental health services in more severe cases.

### Analysis

The characteristics of the sample were described by percentages for the categorical variables and by mean and standard deviation for the continuous variable. Prevalence of SDE and MDE were estimated for the full sample and according to the study variables. Statistically significant differences were estimated using the χ2 test. Additionally, differences in perceived social support scores according to diagnosis criteria were examined by one-way ANOVA. Multinomial logistic regression was used to examine social and economic factors related to SDE and MDE. The dependent variable was the diagnosis criteria for depression: No depression (ND), SDE and MDE, with ND being the reference group. The independent variables were sex, age, indigenous ethnicity, lives with, parenthood, economic quintile, economic difficulties due to the COVID-19 pandemic, inadequate study conditions, social group membership, and perceived social support. All the independent variables examined were entered simultaneously into the regression model in order to observe the effects of each social and economic factor adjusted for the effect of the other independent variables. Relative risk ratios (RRR) were reported for each independent variable in the multinomial logistic model. The analyses were carried out using Stata 16.

## Results

### Characteristics of the Sample

The characteristics of the sample are shown in the second column of Table 1 (Total column). The participants were mostly females aged 20 to 24 years. Approximately one fifth of the students reported having indigenous ethnicity and most of them reported living with one or both parents and had no children. More than half of the students belonged to the poorest quintiles of the population. Only two students belonged to quintile I, so they were grouped together with quintile II. Half reported economic difficulties due to the COVID-19 pandemic and about one-third reported inadequate conditions for studying. About a quarter of the students participated in some social group and their mean perceived social support scores was 37.2 points.

**Table 1:** Prevalence of SDE and MDE by social and economic factors.

	**Total** **(*n* = 1,577)**	**ND** **(*n* = 842)**	**SDE** **(*n* = 226)**	**MDE** **(*n* = 509)**	***p*-value**
Total	100	53.4	14.3	32.3	—
Sex					<0.001
Male	35.4	60.6	13.6	25.8	
Female	64.6	49.4	14.7	35.9	
Age					0.447
17–19	21.6	56.3	12.9	30.8	
20–24	61.4	51.6	14.8	33.6	
25 years or more	16.9	56.2	14.6	29.2	
Indigenous ethnicity					0.316
No	77.7	54.0	14.7	31.3	
Yes	22.3	51.4	13.1	35.5	
Lives with					0.008
Mother, father, or both	70.3	56.2	13.9	29.9	
Other family members	15.5	45.9	14.3	39.8	
Other	14.3	47.6	16.4	36.0	
Parenthood					0.464
No	96.4	53.1	14.4	32.5	
Yes	3.6	61.4	12.3	26.3	
Economic quintile					<0.001
I-II	52.5	52.7	12.8	34.5	
III	23.1	50.4	14.8	34.8	
IV	13.6	52.8	15.0	32.2	
V	10.8	64.1	20.0	15.9	
Economic difficulties due to COVID-19					<0.001
No	49.4	60.5	16.0	23.5	
Yes	50.6	46.5	12.7	40.9	
Inadequate study conditions					<0.001
No	63.9	57.8	15.0	27.2	
Yes	36.1	45.5	13.2	41.3	
Social group membership					0.002
No	73.7	50.8	15.5	33.7	
Yes	26.3	60.7	11.1	28.2	
Perceived social support*	37.2 ± 10.4	40.2 ± 9.6	36.3 ± 9.7	32.5 ± 10.3	<0.001

*ND, No depression; SDE, Subthreshold depressive episode; MDE, Major depressive episode.*

^*^Mean and standard deviation.

### Prevalence of SDE and MDE

The prevalence of SDE and MDE were 14.3 and 32.3%, respectively (Table 1). Statistically significant differences in prevalence were observed according to sex, lives with, economic quintile, economic difficulties due to the COVID-19 pandemic, inadequate study conditions, and belonging to a social group. The prevalence of MDE was higher in the following groups: females, students who did not live with their parents, who belonged to the lowest economic quintiles, who reported economic difficulties due to the pandemic, who reported inadequate study conditions, and who did not belong to a social group. In contrast, the prevalence of SDE in relation to each variable was close to that observed in the full sample, except for those who belonged to quintile V (highest income group), who showed a higher prevalence of SDE and a lower prevalence of MDE. No statistically significant differences were observed according to age, indigenous ethnicity, or parenthood, although a slightly higher percentage of MDE was observed in students between 20 and 24 years of age, of indigenous ethnicity, and those who were parents. Additionally, it was observed that participants with SDE and MDE had lower perceived social support scores (*p* < 0.001), with the lowest scores of the three groups being those of the students with MDE.

### Social and Economic Factors Associated With SDE and MDE

Table 2 shows the results of the multinomial regression model that accounts for the social and economic factors associated with SDE and MDE. Regarding the variables associated with SDE using ND as reference, respondents affiliated with a social group exhibit a 39% reduction in relative risk (RR) for SDE; also, for each point on the perceived social support scale, there is a 4% reduction in the RR for SDE, keeping constant the other social and economic variables of the model. The rest of the variables did not exhibit a statistically significant relationship with SDE.

**Table 2:** Social and economic factors associated with SDE and MDE.

	**SDE** **(*****n*** **=** **226)**			**MDE** **(*****n*** **=** **509)**		
	**RRR**	**95% CI**	***p*-value**	**RRR**	**95% CI**	***p*-value**
Female (ref = male)	1.33	0.97–1.84	0.077	1.52	1.17–1.97	0.002
Age (ref = 25 years or more)						
17–19	0.82	0.49–1.39	0.460	1.02	0.67–1.56	0.910
20–24	1.04	0.68–1.60	0.852	1.27	0.89–1.80	0.189
Indigenous ethnicity (ref = no)	0.98	0.67–1.43	0.923	1.07	0.80–1.42	0.647
Lives with (ref = mother, father, or both)						
Other family members	1.34	0.87–2.07	0.190	1.57	1.12–2.19	0.009
Other	1.49	0.96–2.31	0.077	1.39	0.97–1.99	0.073
Parenthood (ref = no)	0.63	0.26–1.53	0.310	0.44	0.22–0.89	0.023
Economic quintile (ref = V)						
I–II	0.66	0.41–1.05	0.078	1.65	1.01–2.68	0.046
III	0.83	0.50–1.38	0.466	1.98	1.18–3.32	0.010
IV	0.92	0.53–1.62	0.780	2.38	1.37–4.15	0.002
Economic difficulties due to COVID-19 (ref = no)	0.96	0.69–1.31	0.777	1.73	1.35–2.23	<0.001
Inadequate study conditions (ref = no)	0.97	0.70–1.37	0.881	1.24	0.96–1.61	0.101
Social group membership (ref = no)	0.61	0.43–0.89	0.009	0.76	0.58–1.01	0.058
Perceived social support	0.96	0.94–0.97	<0.001	0.93	0.92–0.94	<0.001

*SDE, Subthreshold depressive episode; MDE, Major depressive episode; RRR, relative risk ratio; Ref, reference category for categorical variables*.

Regarding the variables associated with MDE using ND as reference (Table 2), females had a 52% increase in the RR for MDE, living with other relatives (with respect to living with one or both parents) led to a 57% increase in the RR for MDE, those who had economic difficulties due to the pandemic had a 73% increase in the RR for MDE, and those from poorer quintiles (with respect to the richest quintile) were between 1.65 and 2.38 more likely to have MDE, controlling for the other variables in the model. On the other hand, those who were parents had a 44% reduction of the RR for MDE, and for every point in the scale of perceived positive social support there was a 7% reduction in the RR for MDE, keeping the other variables in the model constant. Perceived social support was the only variable related to both SDE and MDE. Age, indigenous ethnicity, and having inadequate study conditions were not associated with either SDE or MDE.

## Discussion

The aim of this study was to estimate the prevalence of SDE and MDE and to examine how social and economic factors relate to SDE and MDE in undergraduate students during the COVID-19 pandemic. First, it was observed that almost half of the students met criteria for either SDE or MDE, with MDE being more frequent. A higher prevalence of MDE was observed particularly in females, students who did not live with their parents, who belonged to the lowest economic quintiles, who reported economic difficulties due to the pandemic, who reported inadequate study conditions, and who did not belong to a social group. In contrast, a higher prevalence of SDE and a lower prevalence of MDE was observed in those belonging to the economic quintile V (highest income group).

Our data indicate a prevalence of depression during the pandemic similar to that reported in multiple meta-analyses (24, 26–28). Globally, it can be asserted that the pandemic has worsened the mental health of university students (27). Nevertheless, it is worth bearing in mind the heterogeneity in depression levels among countries (41). Thus, students in countries such as Turkey have exhibited a major impact (42), unlike those in Sweden, for instance, where rates stayed relatively stable (43). Specifically, in Chile, authors have observed an increase in depressive symptomatology relative to prior research, especially among young population (44). Similar results have been reported for other countries in the region (e.g., (45, 46)). As for subthreshold depression, we found no specific references covering the situation of university students during the pandemic. Nevertheless, a Pre-pandemic study in China reported levels higher than those found in this study (47) but similar to those reported by Chilean adolescents (34).

Second, differences were found in how social and economic factors relate to the level of depressive symptoms. Belonging to a social group and perceiving positive social support were the only variables that had a negative association with SDE, controlling for the other social and economic variables. Instead, female sex, poorer quintiles, living with relatives other than parents, and economic difficulties due to the pandemic were positively associated with MDE, while being a parent and perceived positive social support were negatively associated with MDE. These results are partly in line with previous studies, before and during COVID-19, which showed a relation between social and economic factors and depression (14–21, 24, 26, 48) but expand on what has been reported in the literature by showing differences in the association according to the level of depressive symptoms.

Contrary to expectations, the results showed an inverse gradient in the association between income quintile and MDE. We believe that this result could be an effect of the consequences of the pandemic on family income, since by July 2020, 59.4% of households had seen their income decrease and 30.1% of the respondents had lost their jobs due to the pandemic (49), but public policies to help families economically initially focused on the people with the lowest income, with benefits being subsequently extended to the most vulnerable 80% of the population (i.e., quintiles I to IV) (50).

Specifically perceived social support was the only variable related to both SDE and MDE. This variable has been instrumental in reducing the impact of the COVID-19 pandemic on the mental health of university students (29, 48, 51). Also, regarding subthreshold depression, the participants who were affiliated to a social group exhibited less SDE, which is a novel finding. Thus, belonging to a social group can probably enable students to establish social networks with peers, which, in the presence of subclinical depressive symptoms, can enhance their psychological wellbeing during a pandemic. The fact that social group membership was associated with SDE but not with MDE may suggest that at higher symptom severity group membership alone is not a factor that in itself acts as a buffer for MDE. Thus, the social support resources that students with MDE may be needed as a protective element would be based on the perception of effective social support rather than group membership *per se*.

One of the main limitations of this study is its cross-sectional design, which does not make it possible to establish causal relationships between the variables. Also, the response rate obtained and the fact that the sample is from only one university in southern Chile probably means that the results are not representative of the entire university population at the national or regional level. However, the response rate is within the range of response rates (between 7 and 17%) obtained in previous studies using online surveys with university students (3). Furthermore, in this study, the PHQ-9 was used to classify students into ND, SDE, and MDE groups as in previous studies (34, 35), however, this instrument does not offer sufficient grounds for a diagnosis and does not take into consideration the possible impairment caused by the symptoms, so the categories account for the presence of a set of symptoms that occur at a specific time and do not constitute a clinical diagnosis (37). Considering that we did not use a random sampling and that the prevalence of depression may vary according to the time at which the assessment was taken, population differences and instruments used, comparisons between countries should be made with caution.

The results presented confirm that MDE interventions in university settings must consider students' social and economic characteristics. In addition, they support that MDE and SDE prevention must not only target the most economically vulnerable students. Indeed, it is the wealthiest group that exhibited the most SDE, while the poorest 80% of students (quintiles I-IV) exhibited the most MDE. Also, in this context, offices of students' affairs must prioritize initiatives that promote social relationships and institutional support networks in settings that go beyond the merely academic. Specifically, our findings suggest that young people's participation in social groups could operate as a buffer against SDE. SDE exhibited no differences in connection with the variables examined, thus, subthreshold symptoms during that period were common to all groups, encouraging to explore other variables that might be found to be linked to subthreshold depression in the future; and also to explore which possible dimensions of perceived social support are related to different levels of depressive symptoms.

## Data Availability Statement

The raw data supporting the conclusions of this article will be made available by the authors, without undue reservation.

## Ethics Statement

The studies involving human participants were reviewed and approved by Comité de Ética de la Universidad Austral de Chile. The participants provided their written informed consent to participate in this study.

## Author Contributions

AL, RV, JS, ZA, and TB were involved in the study conception. AL and MC were involved in the study design. MB-C, CC-N, MC-M, BG-C, and CH-P were involved in the extraction of data. MC was involved in data analysis. AL and MC were involved in the interpretation of findings and writing of the manuscript. All authors participated in the review of the manuscript, provided critical revisions, and gave the final approval of the version to be published.

## Funding

AL and MC were partially funded by ANID-Millennium Science Initiative Program-NCS2021_081 and ICS13_005. MC received funding from ANID/PFCHA/DOCTORADO NACIONAL/2019-21190859. RV was partially funded by Centro Nacional de Inteligencia Artificial CENIA, FB210017, BASAL, ANID.

## Conflict of Interest

The authors declare that the research was conducted in the absence of any commercial or financial relationships that could be construed as a potential conflict of interest.

## Publisher's Note

All claims expressed in this article are solely those of the authors and do not necessarily represent those of their affiliated organizations, or those of the publisher, the editors and the reviewers. Any product that may be evaluated in this article, or claim that may be made by its manufacturer, is not guaranteed or endorsed by the publisher.
